# A Prospective Observational Study on the Accuracy of Transcutaneous Laryngeal Ultrasonography in Assessing Vocal Cord Mobility before and after Thyroid Surgery

**DOI:** 10.22038/ijorl.2025.86965.3949

**Published:** 2025

**Authors:** Harjinder Singh, Thirugnanasambandam Nelson, Kamal Kataria, Ankita Agarwal, Uttam Kumar Thakur, Arvind Kairo, Hitesh Verma, Shuchita Singh Pachaury, Amarinder Singh Malhi, Yashwant Rathore, Yashdeep Gupta, Shivam Pandey, Rajesh Khadgawat, Shipra Agarwal, Sunil Chumber, Anita Dhar

**Affiliations:** 1 *Department of Surgical Disciplines, All India Institute of Medical Sciences, New Delhi, India.*; 2 *Department of Radiodiagnosis, All India Institute of Medical Sciences, New Delhi, India.*; 3 *Department of Otorhinolaryngology, All India Institute of Medical Sciences, New Delhi, India*; 4 *Department of Endocrinology, All India Institute of Medical Sciences, New Delhi, India *; 5 *Department of Biostastics, All India Institute of Medical Sciences, New Delhi, India.*; 6 *Department of Pathology, All India Institute of Medical Sciences, New Delhi, India.*

**Keywords:** Transcutaneous Laryngeal Ultrasonography, Vocal Cord Function, Laryngoscopy, Thyroidectomy

## Abstract

**Introduction:**

Recurrent Laryngeal Nerve (RLN) injury remains one of the significant complications associated with thyroidectomy, occurring in approximately 1% to 9% of cases. Vocal Cord (VC) function is typically assessed before surgery using laryngoscopy. However, Transcutaneous Laryngeal Ultrasonography (TLUS) has become a non-invasive alternative for evaluating VC mobility. This study was performed to compare the diagnostic accuracy of TLUS with traditional laryngoscopy in assessing vocal cord function in patients undergoing thyroid surgery.

**Materials and Methods:**

A total of 105 patients undergoing hemi- or total thyroidectomy were enrolled in a prospective observational study at a tertiary healthcare facility from October 2022 to June 2024. TLUS was conducted by endocrine surgeons using a Mindray UGW 11 device. VC mobility was categorised as usual (spontaneous, rhythmic, symmetrical movement) or unilateral VC paralysis (asymmetrical or absent movement on the affected side).

**Results:**

In the preoperative setting, TLUS achieved 100% sensitivity, Positive Predictive Value (PPV), and overall diagnostic accuracy. Postoperatively, it maintained a high sensitivity of 99.02%, with specificity reaching 100% and an area under the curve (AUC) of 0.99. The PPV remained at 100%, while the Negative Predictive Value (NPV) was 75%, and the diagnostic accuracy declined slightly to 99.05%. These findings highlight TLUS as a reliable, economical, and patient-friendly modality for evaluating vocal cord mobility in thyroid surgery.

**Conclusion:**

TLUS is an effective non-invasive method for assessing VC function, with high diagnostic accuracy. With further advancements in ultrasound technology and standardized protocols, TLUS can be incorporated into routine clinical practice as a supplement to traditional laryngoscopy techniques. This study supports the use of TLUS as a viable alternative for preoperative and postoperative VC assessment in thyroid surgery patients.

## Introduction

Thyroidectomy is one of the most commonly performed endocrine surgeries globally, primarily undertaken for conditions such as thyroid cancer, benign goiters, and hyperthyroidism (1,2). Recurrent laryngeal nerve (RLN), injury is a well-recognized complication of thyroidectomy, with incidence rates ranging from 1% to 9% (3). RLN damage can lead to vocal cord paralysis, presenting with symptoms such as hoarseness, difficulty swallowing, breathing difficulties, and in severe cases, life-threatening airway obstruction (4,5). Assessment of vocal cord mobility before surgery is conventionally undertaken using laryngoscopic techniques, which include indirect mirror laryngoscopy and flexible fiber-optic laryngoscopy (FFL) (6). 

Although FFL remains the standard approach, it is invasive, may cause discomfort, and requires both specialized equipment and trained personnel resources that may not be accessible in all clinical environments (7).

Transcutaneous laryngeal ultrasonography (TLUS) has emerged as a practical, non-invasive modality for evaluating vocal cord function (8). It is easy to perform, well-tolerated by patients, and removes the need for sedation or radiation exposure, making it appropriate for both pre- and postoperative assessments (9). 

Since its initial application for laryngeal imaging in 1992 (10), TLUS has gained attention as a viable substitute for traditional laryngoscopic evaluations in the perioperative setting. Additionally, intraoperative neuromonitoring (IONM) of the RLN has become increasingly common, wherein low-voltage electrical stimulation of the nerve produces vocal cord muscle contraction. This is detected via electromyographic (EMG) signals, characterized by specific latency and amplitude, along with an audible sound output (6,11). Previous research has demonstrated that TLUS offers high diagnostic accuracy in identifying vocal cord paralysis after thyroid surgeries (12,13). This study was therefore designed to compare the effectiveness of TLUS with laryngoscopy in evaluating vocal cord function in patients undergoing thyroid surgery.

## Materials and Methods

This prospective observational study was conducted at a tertiary care center between October 2022 and June 2024, following approval from the Institutional Ethics Committee. A total of 105 patients presenting to the surgical outpatient department with thyroid disorders requiring either hemithyroidectomy or total thyroidectomy were recruited. All participants underwent a comprehensive clinical assessment, which included a detailed history-taking and a thorough physical examination. Radiological assessment was performed using neck ultrasonography by experienced radiologists, and thyroid function tests were obtained for the biochemical evaluation. Fine-Needle Aspiration Cytology (FNAC) and other relevant diagnostic investigations were conducted with established institutional protocols.

Following confirmation of diagnosis, preoperative evaluation of vocal cord (VC) mobility was performed by otolaryngologists using indirect laryngoscopy. Subsequently, preoperative TLUS was performed by endocrine surgeons, adhering to a standardized protocol. All TLUS examinations were performed using a Mindray UGW-11 ultrasound system equipped with a 12-MHz broadband linear transducer. After application of conductive gel to the anterior neck, the transducer was placed transversely over the thyroid cartilage and advanced in a craniocaudal direction to visualize both the true and false vocal cords.

Successful identification of the vocal cords was confirmed by the clear delineation of three key laryngeal anatomical landmarks: the arytenoids, true vocal cords, and false vocal cords. Vocal cord motion was assessed using two modes:

A) **Passive assessment** – during quiet breathingB) **Active assessment** – while the patient was instructed to phonate or perform the Valsalva maneuver

VC movement was interpreted as follows:

1. **Normal vocal cord function** – characterized by spontaneous, rhythmic, symmetric movement of both cords
2. **Unilateral vocal cord palsy (VCP)** – indicated by either diminished movement or complete immobility on one side

Postoperatively, while extubating the patient, VC mobility was checked using C-MAC Video Laryngoscope and later using TLUS in the ward. Also, the patients were assessed for signs of hypocalcemia and change in voice.

### Statistical-analysis:

  Categorical variables were summarized using frequencies and percentages, while continuous data were expressed as mean ± standard deviation (SD) or as median with interquartile range (IQR: 25th–75th percentile), depending on the data distribution. The inter-rater reliability between preoperative and postoperative TLUS and indirect laryngoscopy (IDL) was evaluated using Cohen’s kappa coefficient to determine the level of agreement. Diagnostic performance metrics for TLUS-including sensitivity, specificity, positive predictive value (PPV), and negative predictive value (NPV)-were calculated using IDL or flexible fiber-optic laryngoscopy (FFL) as the reference standard. Data were initially entered into Microsoft Excel and subsequently analyzed using IBM SPSS Statistics software, version 25.0 (IBM Corp., 

Chicago, IL, USA). A p-value of less than 0.05 was considered indicative of statistical significance.

## Results

  A total of 105 patients scheduled for thyroid surgery were enrolled in the study to assess preoperative and postoperative vocal cord mobility using transcutaneous laryngeal ultrasonography (TLUS).

*Demographic-profile:* The average age of the participants was 41.04 ± 12.7 years, with the majority (26.67%) in the 31–40 year age group. 

 Their ages ranged from 19 to 69 years. The group was predominantly female, including 90 females (85.71%) and 15 males (14.29%) (Table 1).

**Table 1:** Distribution of Patients by Age Group and Gender Undergoing Thyroid Surgery.

**Age Group (Years)**	**Frequency (%)**
19-30	27 (25.71%)
31-40	28 (26.67%)
41-50	24 (22.86%)
51-60	17 (16.19%)
61-69	9 (8.57%)
Mean ± SD	41.04 ± 12.7
Median (IQR)	40 (30-50)
Gender Female	90 (85.71%)
Male	15 (14.29%)
	

 Most individuals (80.95%) had a normal BMI (18.5–22.99 kg/m²). A smaller percentage was overweight (12.38%) or obese (3.81%), with an average BMI of 21.23 ± 1.98. Only 2.86% of participants were underweight. 

Regarding symptoms, all patients presented with thyroid swelling, and 6.67% reported additional symptoms such as hoarseness, dyspnea, or dysphagia. (Table 2).

**Table 2:** Distribution of BMI and Symptoms of patients undergoing thyroid surgeries

**Category**	**Frequency (%)**
Underweight (<18.5 kg/m²)	3 (2.86%)
Normal (18.5-22.99 kg/m²)	85 (80.95%)
Overweight (23-24.99 kg/m²)	13 (12.38%)
Obese (≥25 kg/m²)	4 (3.81%)
Mean ± SD	21.23 ± 1.98
Median (IQR)	21 (19.98-22.09)
Symptoms Thyroid Swelling	105 (100%)
Pressure symptoms (Hoarseness, Dyspnea, Dysphagia)	7 (6.67%)

A) Preoperative Evaluations:

The average lesion size measured by ultrasonography was 3.88 ± 2.19 cm. Lymphadenopathy was present in 22 (21.15%) cases. TIRADS 4 was the most frequent category (33.77%), indicating a higher suspicion of malignancy. 

Fine Needle Aspiration Cytology (FNAC) BETHESDA classification showed that most patients (46.59%) were classified as BETHESDA 2 (benign nodules). Preoperative TLUS and IDL showed 100% agreement in detecting bilaterally mobile vocal cords in all cases (Table 3).

**Table 3:** Preoperative Findings of thyroidectomy patients

**Parameter**	**Frequency (%)**
Lesion Size (USG) Mean ± SD	3.88 ± 2.19 cm
Lymphadenopathy	22 (21.15%)
TIRADS 4 (Most Common)	26 (33.77%)
BETHESDA 2 (Most Common)	41 (46.59%)
Bilateral vocal cords mobility (TLUS & IDL/FFL)	105 (100%)

B) Postoperative Outcomes:

 Following surgery, vocal cord mobility remained intact in 101 (96.19%) cases, while 4 (3.81%) patients showed non-mobile vocal cords. Postoperative voice changes and hypocalcemia were reported in 4 (3.81%) cases each (Table 4).

**Table 4:** Prevalence of Postoperative Complications using TLUS technique

**Parameter**	**Frequency (%)**
Vocal Cord Mobility A) Mobile	101 (96.19%)
B) Not Mobile	4 (3.81%)
Voice Change	4 (3.81%)
Hypocalcemia	4 (3.81%)

The final histopathology evaluation revealed that 66 cases (70.97%) were benign, while 27 cases (29.03%) were malignant. Among the benign lesions, follicular thyroid nodular disease (20.43%), followed by adenomatous goitre (18.28%). Papillary thyroid carcinoma represented the most frequent malignancy pathology, accounting for 13.98% of cases (Table 5).

**Table 5:** Distribution of Histopathological diagnosis following thyroid surgeries

**Histopathological Diagnosis**	**Frequency (%)**
FTND	19 (20.43%)
Adenomatous Goiter	17 (18.28%)
PTC	13 (13.98%)
Lymphocytic Thyroiditis	8 (8.60%)
Benign Cases	66 (70.97%)
Malignant Cases	27 (29.03%)

Postoperative assessment demonstrated a strong level of agreement between TLUS and IDL/FFL, with a Kappa value of 0.852 (p < 0.0001). TLUS demonstrated high sensitivity (99.02%) and specificity (100%), resulting in an overall diagnostic accuracy of 99.05%. The positive predictive value (PPV) was consistently 100%, whereas the negative predictive value (NPV) was observed to be 75% (Table 6).

**Table 6:** Inter-Rater Agreement & Diagnostic Accuracy of TLUS technique for demonstration of VC mobility

**Parameter**	**Preoperative**	**Postoperative**
Sensitivity (95% CI)	100% (96.55-100%)	99.02% (94.66-99.98%)
Specificity (95% CI)	-	100% (29.24-100%)
AUC (95% CI)	-	0.99 (0.96-1.00)
PPV (95% CI)	100% (96.55-100%)	100% (96.41-100%)
NPV (95% CI)	-	75% (19.41-99.37%)
Overall Accuracy	100%	99.05%

## Discussion

Thyroidectomy carries the potential risk of recurrent laryngeal nerve (RLN) injury, which can lead to vocal cord dysfunction and serious complications, including hoarseness, difficulty swallowing, and even airway compromise. Although flexible fiber-optic laryngoscopy (FFL) is considered the gold standard for assessing vocal cord function, transcutaneous laryngeal ultrasonography (TLUS) provides a non-invasive, patient-friendly, and cost-effective alternative (4,5,8). 

Several studies have assessed the diagnostic performance of TLUS in evaluating vocal cord mobility, with reported sensitivity rates of 92% and 88%, and specificity rates of 96% and 94%, respectively (14,15). However, the higher sensitivity (99.02%) and specificity (100%) observed in the present study may reflect improvements in operator training and advancements in ultrasound technology. Dedecjus et al. reported that transcutaneous laryngeal ultrasonography demonstrated a high negative predictive value (NPV) of 98%, which exceeds the NPV observed in the current study (75%) (6). The slightly reduced NPV observed in this study may be due to a more challenging patient group, which includes individuals with anatomical differences or post-surgical scarring.

 Unlike laryngoscopy, which requires specialized equipment and trained personnel, TLUS provides a cost-effective and readily accessible alternative for VC assessment, as demonstrated in the present study. Similarly, Huang et al. emphasized the applicability of TLUS in outpatient and resource-constrained settings, noting its advantage of being performed without the need for sedation or anesthesia (16).

 Previous studies have shown that factors influencing the diagnostic accuracy of TLUS, such as patient anatomy including neck thickness and the presence of large thyroid masses, are critical determinants (17,18). However, the present study achieved higher diagnostic accuracy, suggesting that operator experience and adherence to standardized protocols were essential for optimal outcomes.

Previous studies have shown that factors influencing the diagnostic accuracy of TLUS, such as patient anatomy including neck thickness and the presence of large thyroid masses, are critical determinants (17,18). However, the present study achieved higher diagnostic accuracy, suggesting that operator experience and adherence to standardized protocols were essential for optimal outcomes.

### Challenges and Limitations

Obesity, large goiters, and calcification of the thyroid cartilage can obscure ultrasonographic views, potentially leading to false-negative results. This limitation, also noted by Woo et al., highlights the need for additional diagnostic tools in complex cases (24). To validate these findings and promote TLUS as a standard diagnostic tool, additional large-scale, multicenter studies with diverse patient populations are necessary.

Although the study achieved impressive results, its findings may not be applicable to all clinical settings, especially those with limited access to high-frequency ultrasound equipment or experienced staff operators.

## Conclusion

 Transcutaneous laryngeal ultrasonography (TLUS) is a reliable, cost-effective, non-invasive method that ensures patient comfort for assessing vocal cord movement during thyroid surgery cases. As ultrasound technology advances and examination protocols become more standardized, TLUS can be incorporated into routine clinical practice alongside traditional laryngoscopy techniques. Further research with larger cohorts and diverse patient demographics is needed to improve its clinical usefulness and establish broader applicability.
